# The versatility of algae in addressing the global sustainability challenges

**DOI:** 10.3389/fbioe.2025.1621817

**Published:** 2025-10-01

**Authors:** Bishnu Dev Das, Ajaya Bhattarai

**Affiliations:** ^1^ Department of Botany, Mahendra Morang Adarsh Multiple Campus, Biratnagar (Tribhuvan University), Biratnagar, Nepal; ^2^ Department of Chemistry, Mahendra Morang Adarsh Multiple Campus, Biratnagar (Tribhuvan University), Biratnagar, Nepal

**Keywords:** algae-based applications, biofuels, bioremediation, carbon sequestration, climate change, food security, global sustainability challenges

## Abstract

Algae have developed into a sustainable and adaptable resource that can help with several global issues, such as resource depletion, environmental degradation, food security, climate change, and energy security. It explores the multifaceted potential of algae in addressing key global sustainability challenges—including climate change, resource depletion, environmental pollution, food insecurity, and energy demands—through biotechnological innovations. Chlorella, Nannochloropsis, Botryococcus, and Spirulina demonstrate exceptional efficiency in biomass production, carbon sequestration, nutrient recycling, and bioenergy generation. Objectives of this review include evaluating recent advances in algal-based wastewater remediation, biodiesel production, and circular bioeconomy strategies, with a focus on the integration of industrial waste streams like abattoir wastewater and crude glycerol. Notably, Chlorella sorokiniana has shown high potential for phycoremediation and biodiesel yield when cultivated in abattoir wastewater digestate (AWD), achieving up to 90% BBM replacement with enhanced lipid and carotenoid content. Similarly, the valorization of crude glycerol via microbial and insect-based systems underscores algae’s role in supporting low-carbon bioeconomies. In agriculture, macroalgae such as Asparagopsis taxiformis have significantly reduced enteric methane emissions in livestock, highlighting their utility in climate-smart farming. Despite these advances, the scalability and economic viability of algal technologies remain constrained by high production costs, energy-intensive processing, contamination risks, and regulatory limitations—especially in food and feed sectors. It advocates for targeted research into cost reduction, process optimization, and harmonized policy frameworks to unlock algae’s full potential. By addressing these challenges, algae can become central to sustainable development strategies, enabling effective transitions toward cleaner energy, healthier ecosystems, and resilient food systems.

## Introduction

Algae are a diverse group of photosynthetic organisms that inhabit a wide range of aquatic environments, including freshwater, marine, and brackish ecosystems. They contribute significantly to global carbon fixation and oxygen production, serving as a fundamental component of the Earth’s biosphere. Algae are generally classified into microalgae and macroalgae, both of which have garnered increasing interest for their applications in renewable energy production due to their fast growth rates, high lipid or carbohydrate content, and adaptability to various environmental conditions. Their potential for wastewater treatment, carbon sequestration, and bioproduct generation positions them as key players in sustainable development and the circular bioeconomy (Gaurav et al., 2024; Chai et al., 2021).

Recent classification of algae has significantly advanced due to the application of molecular phylogenetics and integrative taxonomy, incorporating genomic, morphological, and ecological data. Green algae are now divided into two major lineages: Chlorophyta and Streptophyta. Within Chlorophyta, classes such as *Chlorophyceae*, *Ulvophyceae*, and *Trebouxiophyceae* have been refined using chloroplast genome data, as shown by Liu et al. (2020), who proposed an updated taxonomic scheme for the order Chaetophorales. Streptophyte algae, which include *Zygnematophyceae*, *Charophyceae*, and other early diverging green algae, have also undergone revision, with Bierenbroodspot et al. (2024) highlighting their evolutionary closeness to land plants. Ochrophyta, a diverse group of heterokont algae including brown algae (*Phaeophyceae*) and diatoms (*Bacillariophyceae*), has been split into two major clades: Chrysista and Diatomista.

Recent multigene phylogenetic analyses by Thakur et al. (2019) introduced new classes such as *Olisthodiscophyceae* and *Phaeosacciophyceae*. Red algae (Rhodophyta) have also seen taxonomic refinement, with Costa et al. (2016) identifying new families like *Yamadaellaceae* and *Liagoropsidaceae* within *Nemaliales* using chloroplast phylogenomics. Cyanobacteria, traditionally known as blue-green algae, have benefited from an integrative taxonomic approach that combines morphology with 16S rRNA and ITS sequence data, as emphasized by Kaštovský (2023). Overall, the use of integrative taxonomy, including DNA barcoding and whole-genome analysis, has been critical in resolving complex algal lineages and delineating cryptic species (Heethoff et al., 2011).

Advances in molecular biology and genetic sequencing have significantly altered the classification of algae. The use of ribosomal RNA (rRNA) sequencing in phylogenetic research has revealed new details regarding the evolutionary relationships of groupings of algae. Species have often been reclassified as a result of this. For instance, molecular research has shown that certain kinds of green algae are more closely related to terrestrial plants than previously thought, which has altered our understanding of plant evolution (Lewis and McCourt, 2004). Similarly, molecular technology has enabled the discovery of previously unidentified algae species, highlighting the richness and complexity of the algal kingdom.

## Pigments in algae

Algae contain a variety of pigments that enable them to perform photosynthesis effectively in different aquatic environments. These pigments vary significantly across algal classes. In Chlorophyceae (green algae), the principal pigments are chlorophyll *a* and chlorophyll *b*, along with carotenoids such as β-carotene and lutein. These pigments are similar to those found in higher plants, giving the algae a distinct green coloration (Graham et al., 2009). Phaeophyceae (brown algae) contain chlorophyll *a* and *c*, and a dominant xanthophyll called fucoxanthin. Fucoxanthin masks the green of chlorophyll and gives the algae their characteristic brown coloration. It also improves light absorption in the blue-green spectrum, aiding in photosynthesis at deeper water levels (Barsanti and Gualtieri, 2006).

In Rhodophyceae (red algae), chlorophyll *a* is present, but chlorophyll *b* and *c* are absent. Instead, these algae contain water-soluble phycobilin pigments such as phycoerythrin and phycocyanin. Phycoerythrin, in particular, gives red algae their color and enables efficient photosynthesis in deeper or shaded waters by absorbing green and blue light. Carotenoids like β-carotene and zeaxanthin are also present (Lee, 2008). Bacillariophyceae (diatoms) are rich in chlorophyll *a*, *c*, and fucoxanthin, giving them a golden-brown color. Fucoxanthin enhances their ability to capture light efficiently and is one of the major pigments in this group (Andersen, 2005). Cyanophyceae (cyanobacteria or blue-green algae) primarily contain chlorophyll *a* and phycobilins such as phycocyanin and phycoerythrin. These accessory pigments allow cyanobacteria to thrive in a range of light conditions. Unlike most algae, cyanobacteria do not contain chlorophyll *b* (Whitton and Potts, 2012).

Euglenophyceae (euglenoids) possess chlorophyll *a* and *b*, along with β-carotene. Although they share pigment composition with green algae, they differ in storage material and cell structure, storing carbohydrates as paramylon instead of starch (Buetow, 1968). Finally, Dinophyceae (dinoflagellates) contain chlorophyll *a* and *c* and a unique carotenoid called peridinin. This pigment gives them a reddish-brown appearance and contributes to their efficiency in capturing light in marine environments (Riding et al., 2022).

Phaeophyta, or brown algae, are mainly marine due to their large, multicellular nature. Because they contain the pigment fucoxanthin, they have a brownish color. Brown algae are essential to marine ecosystems because they provide a habitat for a diverse range of organisms. The most well-known species include Macrocystis, Fucus, and Saccharina (Huisman et al., 2005). Complex patterns are produced by the unique silica cell walls of unicellular algae called Bacillariophyta (Diatoms). They are present in both fresh and saltwater environments and are among the most important primary producers in aquatic ecosystems. Diatoms play a major role in fixing carbon worldwide. Navicula, Cyclotella, and Pinnularia are notable genera (Wang, Y. et al., 2022). The unicellular chrysophyta (golden algae) contain pigments such as carotenoids and chlorophyll a and c. Although they can be found in marine environments, they are primarily freshwater species. Aquatic food chains depend on species like Ochromonas and Vaucheria, which belong to the chrysophyta (Kapustin, 2024).

The escalating global sustainability challenges—ranging from climate change, food insecurity, environmental pollution, and energy crises—demand innovative, eco-friendly, and resource-efficient solutions. Algae, owing to their rapid growth, diverse metabolic capabilities, and capacity for bioremediation, biofuel production, and carbon sequestration, have emerged as promising candidates in the quest for sustainable alternatives. Recent reviews underscore the potential of microalgae and macroalgae in various domains, including wastewater treatment (Rawat et al., 2011), bioenergy generation (Borowitzka and Moheimani, 2013), nutraceutical production (Bleakley and Hayes, 2017), and carbon capture technologies (Chisti, 2007). However, while these studies emphasize specific applications, there remains a lack of comprehensive integrative analysis that explores the multifunctional role of algae across multiple sustainability fronts simultaneously. Furthermore, scalability, economic feasibility, and regional adaptability of algal technologies have not been sufficiently addressed in the context of global implementation (Ochoa et al., 2020). Current literature also overlooks the socio-political and ecological dimensions of deploying algal systems at large scale. Hence, there exists a critical research gap in synthesizing multidisciplinary evidence to establish algae as a viable keystone solution in the global sustainability framework, particularly with respect to policy integration, life cycle assessment, and interdisciplinary innovation strategies.

## The status of global sustainability of algae its challenges

Algae offer immense potential in biofuels, carbon capture, wastewater treatment, and food systems, yet face significant sustainability challenges. These include high water and energy demands, limited strain optimization, high production costs, and inadequate policy support. Ecological risks from genetically engineered strains and harmful algal blooms also raise public concern. Moreover, a lack of supply chain infrastructure and regulatory consistency hinders scale-up. Despite promising applications, especially in climate resilience and circular bioeconomy, algae’s global impact remains constrained without breakthroughs in cultivation efficiency, policy incentives, and public trust. Bridging these gaps is vital to fully realizing algae’s role in sustainable development. Here are some essential points that further enrich the discussion of the global sustainability challenges of algae, categorized under relevant themes.

### Scope of algae cultivation and biodiversity constraints

Algae—including microalgae and macroalgae comprise a highly diverse group of photosynthetic organisms, numbering between 200,000 to millions of species (Occhipinti et al., 2023). This biodiversity presents both opportunities and constraints. While extremophile strains offer cultivation advantages in harsh environments, most algae strains remain under-characterized, and regulatory frameworks permit industrial use of only a few, creating bottlenecks in innovation and scale-up (Occhipinti et al., 2023). This imbalance between biodiversity potential and industrial deployment risks limiting large-scale sustainability impacts.

### Environmental and resource pressure

Algal cultivation is resource-intensive, especially in terms of water, nutrients, and energy. Water demands for biofuel-producing strains reach 600–1,900 L per liter of fuel, even when using wastewater or saline water (Alazaiza et al., 2023). Energy inputs for photobioreactors further reduce their environmental benefit, with eutrophication and harmful algal blooms also posing risks if cultivation systems fail (Schischke et al., 2023). These challenges question the net sustainability of algal systems unless resource-efficient, integrated solutions are developed.

### Economic barriers and technological hurdles

High costs associated with harvesting, cultivation systems, and growth media limit algae’s commercial viability. Media recycling is promising, but remains exploratory (Arora et al., 2023). Algal biofuel economic feasibility remains two decades away from maturity, necessitating multibillion-dollar investments and breakthroughs in yield and processing efficiency (Schischke et al., 2023). The disparity between research enthusiasm and real-world economic constraints slows adoption in energy, feed, and biochemical markets.

### Environmental services: carbon sequestration and wastewater treatment

Microalgae offer powerful ecosystem services: CO_2_ fixation up to 50 times faster than terrestrial crops, integration into wastewater treatment, and conversion of nutrients into biomass for valuable compounds (Srimongkol et al., 2022; Schipper et al., 2023). Algal consortia have treated industrial wastewater and remediated heavy metals and organics (Walters et al., 2024). However, scaling these solutions from lab to industry remains restricted by cost and infrastructure limitations.

### Social perceptions and regulatory challenges

Public acceptance—especially around genetically engineered (GE) algae—is mixed. Concerns center on escapes from open systems, ecological risks such as harmful blooms, and insufficient regulations (Calatrava et al., 2024). Stakeholders advocate transparent communication regarding GE benefits and risks, deployment of closed-loop systems, and robust environmental risk assessments (Calatrava et al., 2024). Without increased public and policy confidence, investments may lag even as technical innovations advance.

### Land use and competition with agriculture

While algae do not require arable land, large-scale cultivation still demands space. Coastal areas or deserts are often considered, but they may overlap with conservation zones or local livelihoods (Ghosh et al., 2021). Moreover, the infrastructure (e.g., raceways, photobioreactors) could disrupt fragile ecosystems. Sustainable land-use planning is essential to balance algae production and ecological integrity.

### Supply chain and infrastructure limitations

The lack of a robust supply chain—from seed culture to large-scale harvesting, dewatering, and product refinement—remains a bottleneck. Transportation of wet biomass is costly, and decentralized production is often needed, which increases complexity and cost (Carina et al., 2021). Cold chain logistics for high-value products (like omega-3 oils or pigments) further increase emissions unless localized systems are optimized.

### Policy and investment gaps

Algae biotechnology lacks consistent regulatory frameworks across regions. Incentives like carbon credits, renewable fuel subsidies, or wastewater treatment offsets are either inadequate or poorly enforced (Moshood et al., 2021). Inconsistent government backing discourages private investment, and algae startups face high capital expenditures without clear long-term policy guarantees.

### Genetic engineering and strain optimization

Modern CRISPR-based gene editing tools offer the potential to improve yield, lipid productivity, and stress tolerance in algae. However, regulatory resistance and ecological concerns about releasing genetically modified algae hinder their deployment. Without public trust and international consensus on bioengineered strains, breakthroughs in strain optimization may remain underutilized (Dhokane et al., 2023).

### Life cycle and end-of-life impacts

Sustainability assessments often overlook the complete life cycle of algal products. The disposal or reuse of algal residues (post oil-extraction) can pose environmental risks if not managed properly. Residues may carry heavy metals or pathogens from wastewater-grown algae. Strategies for composting, anaerobic digestion, or bioplastics production from algal waste are under development but not yet widely adopted (Lopez-Tenllado et al., 2021).

Among the major sustainability problems caused by the world’s expanding population, industrialization, and environmental degradation are climate change, resource depletion, and food insecurity. Algae have emerged as a potential biological resource that can assist with various sustainability challenges due to their numerous applications in food security, wastewater treatment, carbon sequestration, and biofuel generation (Razzak et al., 2013). Because of their high photosynthetic efficiency, rapid growth rates, and capacity to adapt to a variety of climatic conditions, algae offer a desirable alternative to conventional resources (Wijffels and Barbosa, 2010). Additionally, algae play a vital role in wastewater treatment by efficiently removing nitrogen, phosphorus, and heavy metals from wastewater and producing biomass for bioenergy or agricultural use (Li et al., 2019).

Looking forward, the future of algae sustainability lies in overcoming these economic and regulatory bottlenecks through technological innovation and policy support. Biorefinery approaches, where multiple high-value products are extracted from algae, can improve economic returns and resource efficiency. Advancements in synthetic biology, low-cost cultivation technologies, and integration with existing industrial systems are likely to enhance the scalability of algae solutions (Wertz and Perez, 2023). Public-private partnerships and government incentives will be vital in supporting research and accelerating market adoption.

## Potential application of algae

**FIGURE 1:**
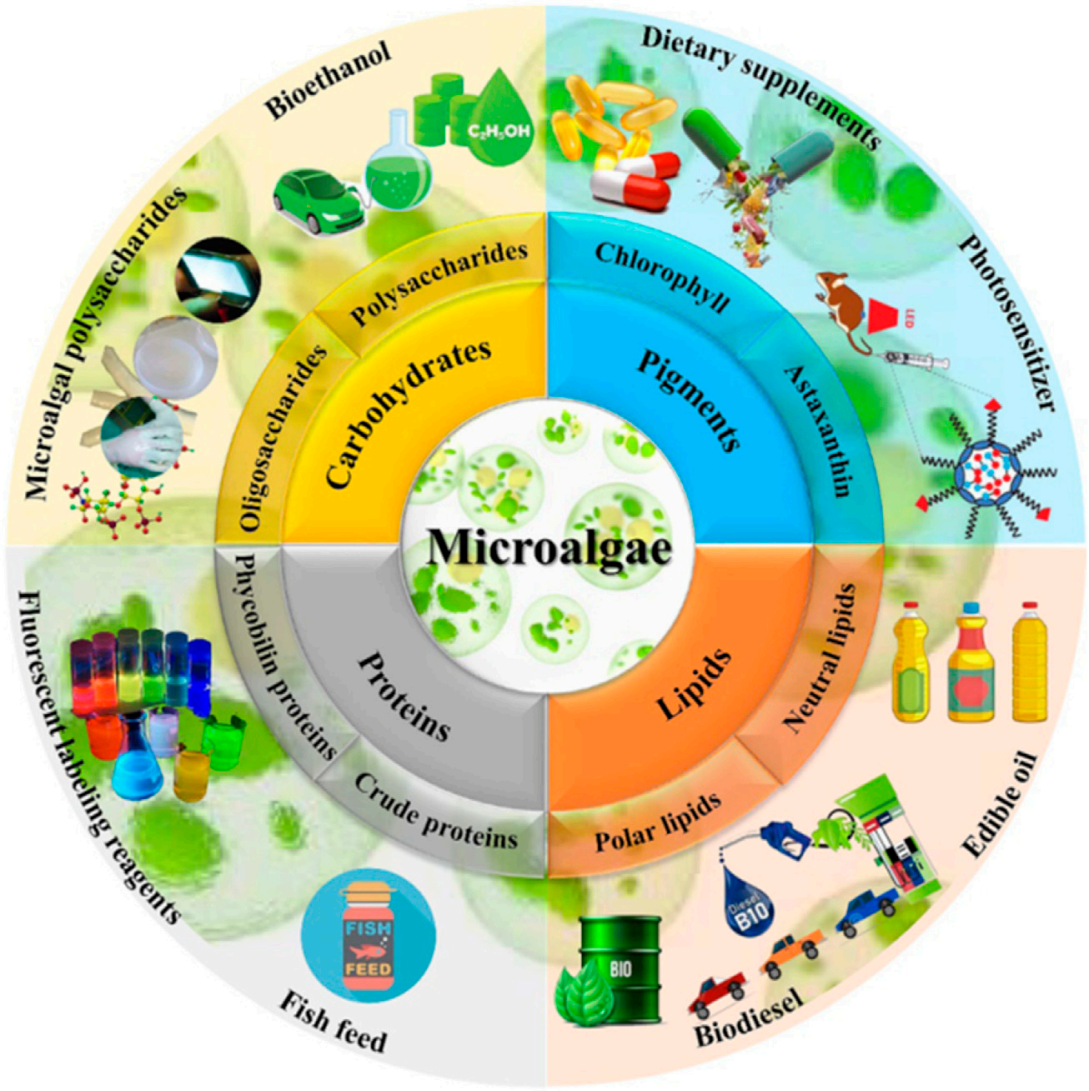
Potential application of microalgae in various fields (Hoang et al., 2022).

Algae contribute to climate change mitigation by sequestering carbon, as they absorb significant amounts of atmospheric CO_2_, thereby reducing greenhouse gas concentrations (Markou and Georgakakis, 2011). Furthermore, items made from algae, such as food supplements high in protein and biodegradable plastics, offer long-term answers to environmental pollution and food security (Henrikson, 2011). Algae have enormous potential to address global sustainability issues because of their versatility. Their broad use in a variety of sectors, including as agriculture, energy, and environmental management, can greatly aid in the accomplishment of the Sustainable Development Goals (SDGs) of the UN (Benemann, 2013). The contribution of microalgae to the circular economy and waste-to-energy plan, while discussing possible industrial and commercial biofuel production methods. Future studies should concentrate on creating new species, integrating biomass pretreatment methods, and streamlining the processes involved in producing biofuel (Hoang et al., 2022). However, overcoming technological, financial, and policy-related obstacles is necessary for the widespread commercial adoption of algae-based solutions. Unlocking algae’s full potential applications for the future requires ongoing research and innovation (Figure 1).

## Applications of different species of algae in biofuel production

Recent studies highlight the promising role of various algal species in sustainable biofuel production. *Neochloris oleoabundans*, a microalga, has attracted attention for biodiesel production due to its high lipid accumulation under nitrogen-limited conditions (Pruvost et al., 2009). Similarly, *Chlamydomonas reinhardtii* is being engineered to enhance starch content, making it a suitable feedstock for bioethanol production (Wang K. et al., 2022). *Spirulina platensis*, beyond its nutritional value, has been investigated for biogas generation through anaerobic digestion, showing improved methane yields when co-digested with agricultural residues (Varol and Ugurlu, 2015). For macroalgae, *Cladophora glomerata*, a freshwater green alga, has been explored for bioethanol production owing to its cellulose-rich biomass that can be efficiently hydrolyzed (Boonprab et al., 2017). Additionally, *Padina tetrastromatica*, a brown macroalga, has demonstrated potential for biodiesel production, with significant lipid content suitable for transesterification (Govindan et al., 2022). These studies reflect the growing interest in diverse algal species as renewable biofuel resources, contributing to a circular bioeconomy.

Table 1 highlights the diverse applications of different algal species in biofuel production, emphasizing their types, biofuels produced, and notable features. *Chlorella vulgaris* ESP-31, a microalga, is used for bioethanol production due to its high carbohydrate content and ability to grow in unsterilized swine wastewater, resulting in high ethanol yield (Acebu et al., 2022). *Nannochloropsis oculata*, another microalga, produces bioethanol from defatted biomass, with mixotrophic cultivation enhancing fermentable carbohydrate levels (Fetyan et al., 2021). *Botryococcus braunii* is noted for biodiesel production, with lipid content reaching up to 75% of its dry weight owing to its hydrocarbon-rich composition (Nazloo et al., 2024). *Scenedesmus obliquus*, categorized as macroalgae in the table, supports dual production of biodiesel and bioethanol due to its high lipid and carbohydrate levels (Kim et al., 2020). *Dunaliella salina* is a salt-tolerant microalga valuable for biodiesel production, rich in β-carotene and lipids (Dali et al., 2021). *Porphyridium cruentum*, a red microalga, provides both biohydrogen and biodiesel thanks to its carbohydrate and pigment content (Markou and Nerantzis, 2013). Among macroalgae, *Gracilaria edulis* is polysaccharide-rich and achieves high bioethanol fermentation efficiency (Ochoa et al., 2020), while *Ulva lactuca* is a fast-growing green algae used for bioethanol and biogas production (Gengiah et al., 2023). Lastly, *Sargassum* spp., invasive brown algae rich in alginate and carbohydrates, are suitable for conversion into both biogas and bioethanol (Orozco-González et al., 2022). Together, these species illustrate the potential of algae as versatile feedstocks for various biofuels.

**TABLE 1:** Applications of different species of algae in biofuel production.

Algal species	Type	Biofuel type	Notable features	Citation
*Chlorella vulgaris* ESP-31	Microalgae	Bioethanol	High carbohydrate content; cultivated in unsterilized swine wastewater; high ethanol yield	Acebu et al. (2022)
*Nannochloropsis oculata*	Microalgae	Bioethanol	Defatted biomass; mixotrophic cultivation increased fermentable carbohydrates	Fetyan et al. (2021)
*Botryococcus braunii*	Microalgae	Biodiesel	Produces hydrocarbons (botryococcenes); lipid content up to 75% dry weight	Nazloo et al. (2024)
*Scenedesmus obliquus*	Macroalgae	Biodiesel, Bioethanol	High lipid and carbohydrate content; suitable for dual fuel production	Kim et al. (2020)
*Dunaliella salina*	Microalgae	Biodiesel	Salt-tolerant; contains β-carotene and high lipid content	Dali et al. (2021)
*Porphyridium cruentum*	Red microalgae	Biohydrogen, Biodiesel	Rich in carbohydrates and pigments	Markou and Nerantzis (2013)
*Gracilaria edulis*	Macroalgae	Bioethanol	Polysaccharide-rich red macroalga; high fermentation efficiency	Yang et al. (2020)
*Ulva lactuca*	Microalgae	Bioethanol, Biogas	Abundant coastal green algae; fast-growing; rich in carbohydrates	Gengiah et al. (2023)
*Sargassum spp.*	Microalgae	Biogas, Bioethanol	Invasive brown algae; rich in alginate and carbohydrates; suitable for bioconversion	Orozco-González et al. (2022)

The study found that replacing up to 90% of Bold’s basel medium (BBM) with abattoir wastewater digestate (AWD) supports algal growth with minimal impact on biomass yield. This approach achieved the highest nutrient removal efficiency and produced high-quality biodiesel. Although all AWD treatments met irrigation water quality standards, AWD90 required slight post-treatment. Higher AWD ratios boosted lipid and carotenoid content in the algae, enhancing biodiesel potential. The research highlights *Chlorella sorokiniana*’s promise for AWD phycoremediation and biomass production, supporting sustainable, waste-to-resource biotechnologies. Further research is needed on scalability, industrial challenges, and economic feasibility for large-scale use in abattoirs (Elsayed et al., 2024). Microorganisms like *Escherichia coli* and *Klebsiella pneumoniae* can convert crude glycerol into ethanol, volatile fatty acids, and hydrogen. Additionally, oleaginous organisms such as yeast, microalgae, and saprophagous insects can transform it into bio-lipids, supporting low-carbon bioeconomy goals. Co-valorization strategies with domestic wastewater or lignocellulosic hydrolysates enhance lipid production. Notably, black soldier fly larvae efficiently convert waste glycerol and protein-rich materials into biodiesel, animal feed, and fertilizers (Elsayed et al., 2023).

Microalgae such as *Chlorella vulgaris*, *Nannochloropsis oceanica*, and *Scenedesmus obliquus* possess high lipid content and fast growth rates, making them ideal candidates for biodiesel. Recent developments in genetic engineering and metabolic pathway optimization have significantly improved lipid productivity and conversion efficiencies, making algal biofuels a technically viable option (Wang et al., 2024). Nevertheless, the production cost of algal biofuel remains significantly higher than that of fossil fuels, impeding commercial competitiveness (Caporgno and Mathys, 2018).

## Contribution of different species of algae in climate change mitigation through carbon sequestration

Another major advantage of algae is their capacity for carbon dioxide (CO_2_) sequestration. Algae can absorb CO_2_ at rates exceeding terrestrial plants, offering a promising solution for mitigating greenhouse gas emissions from power plants and other industrial sources (Srimongkol et al., 2022).

Climate change, primarily driven by excessive carbon dioxide (CO_2_) emissions from industrial activities and fossil fuel combustion, poses a significant threat to global ecosystems. Algae play a major role in climate change mitigation by capturing and storing atmospheric CO_2_ through photosynthesis, a process known as biological carbon sequestration (Markou and Georgakakis, 2011). Microalgae, such as *Chlorella vulgaris*, *Nannochloropsis sp.*, and *Scenedesmus obliquus*, exhibit high photosynthetic efficiency and can capture CO_2_ from industrial flue gases, thereby reducing emissions (Wang et al., 2008). *Chlorella vulgaris*, for example, has been reported to fix up to 1.83 g of CO_2_ per gram of biomass, making it one of the most efficient species for carbon sequestration (Brennan and Owende, 2010). Additionally, microalgae can be cultivated in photobioreactors or open ponds using wastewater, further enhancing their sustainability by integrating CO_2_ capture with wastewater treatment (Razzak et al., 2013). Macroalgae, or seaweeds, such as *Macrocystis pyrifera* (giant kelp), *Sargassum sp.*, and *Laminaria japonica*, play a vital role in oceanic carbon sequestration. These large marine algae absorb CO_2_ during photosynthesis and store carbon in their biomass, which can later be transported to the deep ocean when the algae decay or sink (Duarte et al., 2022). Research suggests that kelp forests can sequester an estimated 173 Tg (teragrams) of CO_2_ annually, highlighting their potential in mitigating climate change (Krause-Jensen and Duarte, 2016).

Certain algae species can be used to produce biochar, a carbon-rich material that can be applied to soil for long-term carbon storage. *Spirulina platensis* and *Chlorella pyrenoidosa* have been investigated for their potential to produce biochar through pyrolysis, thereby locking carbon in stable forms while also improving soil fertility (Lehmann et al., 2006). Additionally, algae-based biofuels provide a sustainable energy alternative that recycles CO_2_ during production, creating a closed carbon loop and reducing net emissions (Wijffels and Barbosa, 2010). Algae-based carbon capture and utilization (CCU) systems have been developed to integrate CO_2_ sequestration with valuable product generation. Species such as *Dunaliella salina* and *Tetraselmis suecica* can thrive in high CO_2_ environments and are utilized in necessary applications like biofertilizers, fodder, and pharmaceutical products. Furthermore, algae can also absorb airborne pollutants like sulfur dioxide (SO_2_) and nitrogen oxides (NOx), which contribute to acid rain and respiratory diseases (Brennan and Owende, 2010). Contributions of different species of algae in climate change mitigation via carbon sequestration are enumerated (Table 2).

**TABLE 2:** Contribution of different algal species in climate change mitigation via carbon sequestration.

Algal species	Type	Carbon sequestration mechanism	Key contribution	Citation
*Chlorella vulgaris*	Microalga	Rapid photosynthetic CO_2_ fixation in PBRs; effective with flue-gas integration	Widely demonstrated in pilot photobioreactors and hybrid scrubber–algae systems for industrial CO_2_ capture and biomass valorization	Prasad et al. (2021)
*Nannochloropsis spp.* (e.g., *N. oceanica*)	Microalga	High photosynthetic/carbon conversion; tolerant to variable CO_2_ and saline conditions	Used in pilot CO_2_ capture + biomass (lipid) production; attractive for biorefinery links	Gaber et al. (2022)
*Scenedesmus obliquus*	Microalga	Flue-gas tolerant biofixation; high areal productivity	Frequently used for industrial CO_2_ biofixation with reported high removal efficiencies in outdoor ponds/PBRs	Li et al. (2023)
*Dunaliella salina*	Microalga (halotolerant)	CO_2_ fixation in high-salinity systems	Suited to saline/brine streams and co-valorization (β-carotene) with promising LCA scenarios for negative CO_2_eq in niche systems	Dali et al. (2021)
*Tetraselmis suecica*	Microalga	High carbon uptake; tolerant to high CO_2_; robust biomass yields	Used in high-rate systems and integrated CCU schemes (fertilizer/feed co-products)	Srimongkol et al. (2022)
*Botryococcus braunii*	Microalga	Accumulates hydrocarbon-like lipids (botryococcenes) from CO_2_	High hydrocarbon yields → direct liquid hydrocarbon precursors; potential for CO_2_→fuel pathways	Nazloo et al. (2024)
*Ulva spp*. (sea lettuce)	Macroalga	High coastal biomass production; biochar/soil application for durable carbon storage	Harvested biomass can be converted to biochar or soil amendments for long-term carbon sequestration and eutrophication control	Pizarro-Loaiza et al. (2021)
Saccharina/kelps (e.g., *S. latissima*)	Macroalga (kelp)	Rapid coastal growth; potential export/burial of biomass (blue carbon)	Kelp farms produce large biomass rapidly, potential CDR pathways include sinking, burial or durable product manufacture (verification needed)	Duarte et al. (2022)
*Sargassum spp*.	Brown macroalga	High productivity and export of particulate/dissolved carbon	Large floating mats export C to deeper waters or support valorization routes, but permanence and ecological trade-offs are active research areas	Wang et al. (2023)

Algae play a crucial role in environmental sustainability through wastewater treatment. Microalgae can effectively remove nitrogen, phosphorus, and heavy metals from municipal and industrial wastewater, converting pollutants into valuable biomass. This biomass can be further used for bioenergy or as biofertilizer, thereby closing nutrient loops (Li et al., 2023). However, inconsistent wastewater composition and the need for pretreatment in some cases raise safety and efficiency concerns, particularly when biomass is reused in food or agriculture.

The contribution of algae to climate change mitigation is multifaceted, primarily through their efficient carbon sequestration capabilities. Algae absorb large amounts of CO_2_ during photosynthesis, which helps reduce atmospheric greenhouse gases. Microalgae, in particular, exhibit rapid growth rates and high carbon fixation efficiency, making them promising candidates for bio-based carbon capture technologies (Severo et al., 2019; Wongsodiharjo and Ismail Masjud, 2024). Additionally, algae-based biofuels provide a renewable alternative to fossil fuels, lowering net carbon emissions when used as energy sources (Wang et al., 2024). Algal bioremediation of wastewater further reduces nutrient runoff that can exacerbate climate-related issues such as eutrophication (Iakovidou et al., 2024). Collectively, these applications position algae as a vital tool in integrated strategies to mitigate climate change.

## Contribution of different species of algae in food security

Food security is a critical global challenge, exacerbated by population growth, climate change, and resource depletion. Algae, both microalgae and macroalgae, have emerged as a sustainable and nutrient-rich solution to enhance food security by providing essential proteins, lipids, vitamins, and bioactive compounds (Pulz and Gross, 2004). Microalgae are abundant in remarkable proteins, carrying all essential amino acids required for human nutrition. Species such as *Spirulina platensis*, *Chlorella vulgaris*, and *Dunaliella salina* are widely recognized as alternative protein sources that can complement or replace conventional animal-based proteins (Becker, 2007). *Spirulina platensis* has a protein content of up to 70% of its dry weight and is considered one of the most complete plant-based protein sources (Habib et al., 2008). Additionally, *Chlorella vulgaris* is packed with essential nutrients, including vitamins (B12), iron, and omega-3 fatty acids, making it a valuable food supplement (Nova et al., 2020).

Macroalgae, or seaweeds, such as *Saccharina japonica* (kelp), *Porphyra spp.* (nori), and *Ulva lactuca* (sea lettuce), have been staples in many Asian diets for centuries. These species are rich in dietary fiber, iodine, calcium, and polysaccharides such as alginate, fucoidan, and carrageenan, which offer significant health benefits (Holdt and Kraan, 2011). *Porphyra spp.*, used in sushi wraps, is particularly valued for its high protein and vitamin B12 content, making it an important food source for vegetarian and vegan diets (Kaur et al., 2023). Algae are increasingly being used as feed ingredients for livestock and aquaculture, enhancing food production sustainability. *Nannochloropsis sp.*, *Tetraselmis suecica*, and *Isochrysis galbana* are commonly used in aquafeeds due to their high levels of omega-3 fatty acids, which improve fish health and nutritional quality (Hemaiswarya et al., 2010). Additionally, incorporating *Spirulina* and *Chlorella* into poultry and cattle feed has been shown to enhance animal growth, immune function, and meat quality while reducing reliance on conventional feed sources (Becker, 2007). Algae are also increasingly being incorporated into agriculture and animal husbandry. In particular, red macroalgae such as *Asparagopsis taxiformis* have been shown to drastically reduce enteric methane emissions when added to cattle feed. A pilot project in Hawaii reported a methane reduction of over 77%, marking a significant breakthrough in climate-friendly agriculture (Associated Press, 2023).

**FIGURE 2:**
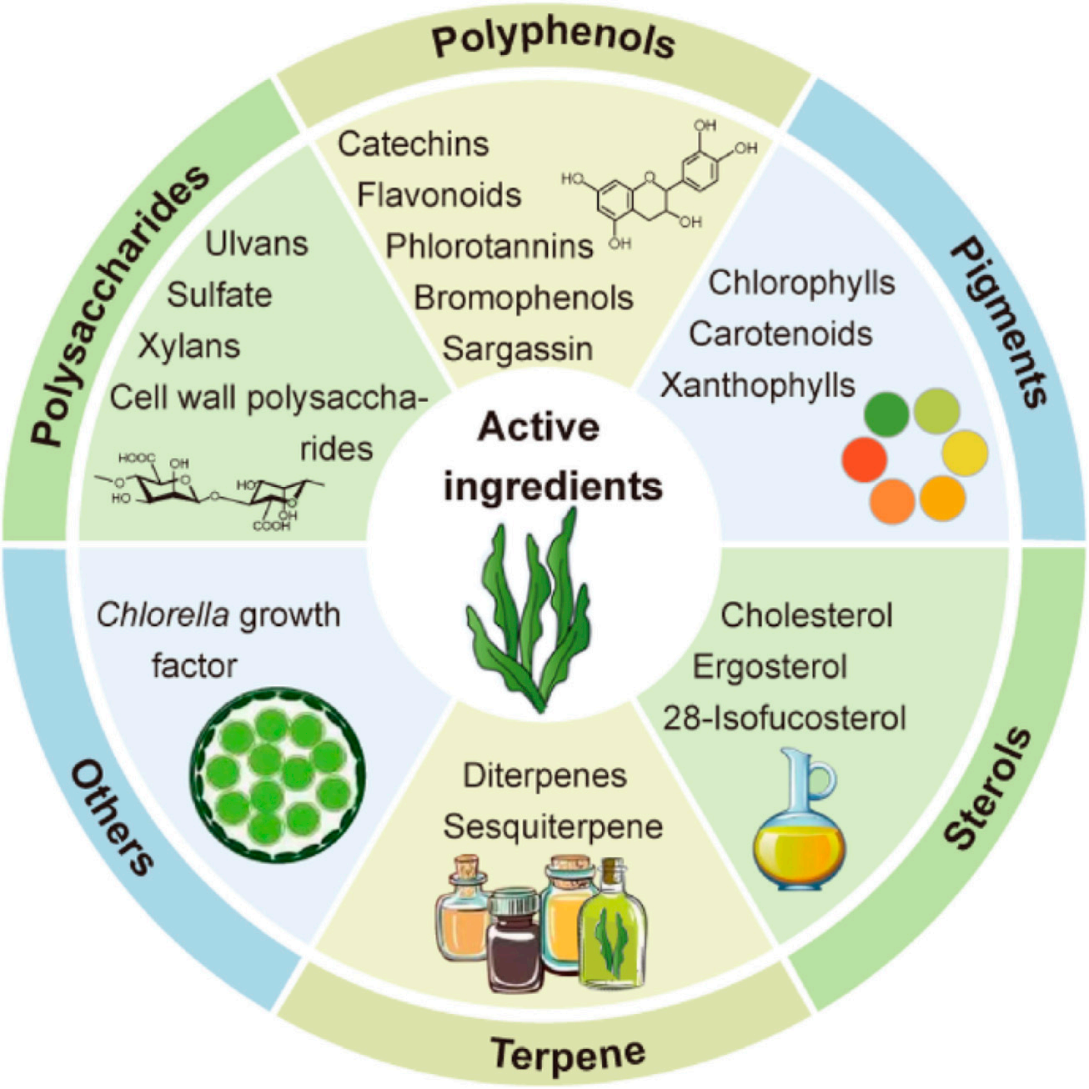
Nutrients and bioactive substances present in different green seaweeds (Heethoff et al., 2011).

The most prevalent macroseaweed species, green seaweed, is a valuable marine biological resource. Numerous amino acids, fatty acids, dietary fibers, polysaccharides, polyphenols, pigments, and other active ingredients are abundant in it. These components are essential for a number of biological functions, including immunoregulation, anti-inflammatory response, and antioxidant activity. The exploration and use of green seaweeds for increased economic value has surged in recent years due to increased awareness of marine resources (Figure 2).

Algae are rich in bioactive compounds, including antioxidants, polyunsaturated fatty acids (PUFAs), and polysaccharides, which contribute to human health and disease prevention. *Dunaliella salina* is a major source of beta-carotene, a powerful antioxidant that supports vision and immune health (Borowitzka, 2013). *Haematococcus pluvialis* produces astaxanthin, a potent anti-inflammatory compound used in nutraceuticals and dietary supplements (Guedes et al., 2011). Algae-based biofertilizers, derived from species such as *Ascophyllum nodosum* and *Sargassum sp.*, contribute to sustainable agriculture by enhancing soil fertility, increasing crop yields, and reducing dependency on chemical fertilizers (Khan et al., 2009). These seaweed extracts contain growth-promoting hormones, minerals, and polysaccharides that improve plant resistance to environmental stresses, supporting more resilient food production systems (Table 3).

**TABLE 3:** Contribution of algal species to food security.

Algal species	Type	Nutritional components	Food security contribution	Citation
Microalgae (mixed species for alternative protein)	Microalgae	Protein, essential amino acids, functional lipids	Emerging vertical/industrial systems can produce concentrated alternative proteins with smaller land footprints	Carina et al. (2021)
*Chlorella vulgaris*	Microalga	Protein, vitamins, essential fatty acids, pigments	Common food supplement; used in fortified foods and as a micro-ingredient for nutrition density	Acebu et al. (2022)
*Nannochloropsis spp.*	Microalga	EPA and other marine omega-3s, protein	Source of marine omega-3s for human food and aqua feed, lowering pressure on fish stocks	Fetyan et al. (2021)
*Haematococcus pluvialis*	Microalga	Astaxanthin (high-value carotenoid)	Nutraceutical and feed additive improving food value chains (aquaculture pigmentation, antioxidant benefits)	Mota et al. (2021)
*Dunaliella salina*	Microalga	β-carotene, provitamin A precursors	Used as a natural pro-vitamin A source and colorant-relevant in micronutrient interventions	Dali et al. (2021)
*Ulva spp.*/*Ulva lactuca*	Macroalga	Dietary fibre, protein, minerals (iodine, Ca, Mg)	Edible seaweed used directly in foods and as animal feed; supports coastal food systems and local nutrition	Gengiah et al. (2023)
Porphyra*/*Pyropia spp. (nori)	Red macroalga	High protein, B-vitamins (B12/analogues), mineral content	Staple seaweed in many diets-demonstrable roles in local food security and micronutrient supply	Heethoff et al. (2011)
*Gracilaria spp./Gracilaria edulis*	Red macroalga	Polysaccharides (agar), sugars, protein	Edible seaweed and hydrocolloid source, supports local food industry and fermentation for ethanol/food ingredients	Vijay Anand et al. (2018)
*Sargassum spp*. (selected uses)	Brown macroalga	Carbohydrates, alginate, minerals	When harvested (e.g., beach cast), can be up cycled into feed/fertilizer or processed for food additives, useful in coastal food security and waste valorization	Orozco-González et al. (2022)

## Contribution of different species of algae in environmental pollution mitigation

Environmental pollution, including water, air, and soil contamination, is a major global challenge. Algae, both microalgae and macroalgae, have shown remarkable potential in mitigating pollution through bioremediation, biofiltration, and pollutant sequestration. Different algal species can absorb heavy metals, degrade organic pollutants, and capture excess nutrients, making them valuable tools for environmental cleanup and ecosystem restoration (Rai et al., 2019). Microalgae have been extensively studied for their ability to remove heavy metals such as cadmium (Cd), lead (Pb), arsenic (As), and mercury (Hg) from industrial wastewater. Species like *Chlorella vulgaris*, *Scenedesmus obliquus*, and *Spirulina platensis* exhibit high biosorption capacities, effectively reducing metal toxicity in contaminated water (Mata et al., 2010). The cell walls of these algae contain polysaccharides, proteins, and lipids that bind metal ions, enabling their removal from industrial effluents (Ordóñez et al., 2023). Moreover, *Chlorella vulgaris* has been used in phycoremediation to treat textile and pharmaceutical wastewater, removing dyes and other organic contaminants (Muñoz and Guieysse, 2006). Similarly, *Scenedesmus obliquus* has demonstrated significant potential in treating tannery and paper mill effluents by reducing chemical oxygen demand (COD) and biological oxygen demand (BOD) levels (Kumar and Sweety, 2024). Phycoremediation offers cost reductions and renewable bioenergy options and can remove heavy metals and harmful organic substances without secondary contamination. Algal species like Chlamydomonas, Chlorella, and Scenedesmus are widely used for wastewater treatment. Phycoremediation also produces valuable biomass with high protein and lipid contents, promising applications in biofuel, food, and animal feed industries (Dayana Priyadharshini et al., 2021).

**FIGURE 3:**
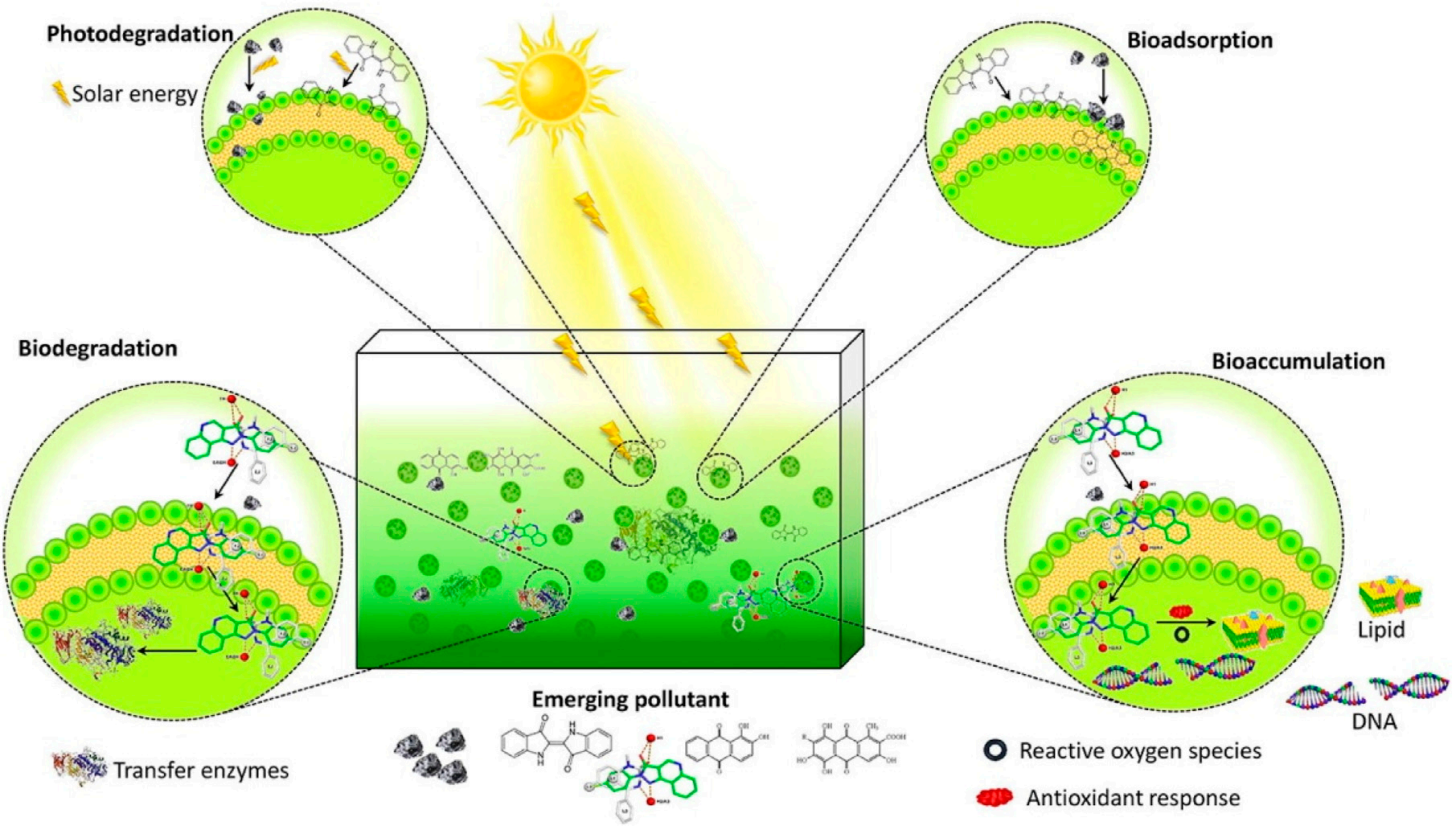
Removing emerging pollutants using algal-bioremediation systems (Gondi et al., 2022).

Eutrophication, caused by excessive nitrogen (N) and phosphorus (P) from agricultural runoff and sewage discharge, leads to algal blooms and oxygen depletion in water bodies. Certain microalgae, such as *Chlorella sp.*, *Nannochloropsis* sp., and *Tetraselmis suecica*, efficiently absorb excess nutrients, prohibiting deleterious algal blooms (HABs) (Cai et al., 2013). Additionally, macroalgae such as *Ulva lactuca* and *Gracilaria sp.* act as biofilters in aquaculture systems, absorbing nitrates and phosphates, thereby reducing eutrophication risks (Neori et al., 2004). Integrated algae-based wastewater treatment systems have been implemented in several regions, demonstrating the ability of algae to recycle nutrients while producing valuable biomass for biofertilizers and biofuels (Park et al., 2011). Certain algae species play a crucial role in air pollution mitigation by capturing carbon dioxide (CO_2_) and other air pollutants from industrial emissions. *Chlorella vulgaris*, *Nannochloropsis sp.*, and *Scenedesmus dimorphus* have been successfully cultivated in photobioreactors for CO_2_ capture from flue gases, significantly reducing greenhouse gas emissions (Wang et al., 2008). Furthermore, algae can also absorb airborne pollutants such as sulfur dioxide (SO_2_) and nitrogen oxides (NOx), which contribute to acid rain and respiratory diseases (Brennan and Owende, 2010). Algal biotechnology offers a low-cost, low-energy solution for wastewater bioremediation. However, commercialization is still in its infancy. Challenges include selecting microalgal species, addressing new contaminants, and operational conditions. Algal bioremediation, combined with current treatment technology, could effectively remove new contaminants (Figure 3).

Algae-based bioremediation is an emerging strategy for degrading persistent organic pollutants (POPs) and pharmaceutical residues in wastewater. Species such as *Chlorella pyrenoidosa*, *Anabaena sp.*, and *Oscillatoria sp.* have shown the ability to break down pesticides, herbicides, and endocrine-disrupting chemicals through enzymatic and photochemical processes (Zahmatkesh et al., 2023). For instance, *Chlorella pyrenoidosa* has been reported to degrade antibiotics such as tetracycline and sulfamethoxazole, reducing their ecological impact on aquatic ecosystems (Li et al., 2022). Algae have been explored as a potential bioremediation tool for cleaning oil spills in marine environments. Moreover, macroalgae such as *Sargassum sp.* have been tested for their ability to absorb oil contaminants from seawater, offering a sustainable and eco-friendly alternative to conventional oil spill cleanup techniques (Ayele et al., 2021). Contributions of different algal species in environmental pollution mitigation are enumerated below (Table 4).

**TABLE 4:** Contribution of algal species in environmental pollution mitigation.

Algal species	Type	Target pollutants	Mitigation mechanism	Key contribution	Citation
*Chlorella vulgaris*	Microalga	N, P, heavy metals, dyes, pharmaceuticals	Bio-sorption, nutrient assimilation, biodegradation (in wastewater PBRs/raceways)	Demonstrated high N/P removal and micro-pollutant attenuation in pilot wastewater and flue-gas integrated systems	Elsayed et al. (2024), Srimongkol et al. (2022)
*Scenedesmus/Desmodesmus spp*. (e.g., *S. obliquus*)	Microalga	N, P, COD, some organics	High nutrient uptake, good biomass yield for co-product recovery	Frequently employed in wastewater nutrient removal with simultaneous biomass production for bio-energy	Kim et al. (2020), Srimongkol et al. (2022)
*Nannochloropsis oculata*/*spp.*	Microalga	Nutrients, hydrocarbons in aquaculture wastewater	Assimilation into biomass; remediation + feed ingredient production	Effective for treating aquaculture effluents while producing feed-quality biomass	Sutherland et al. (2014), Fetyan et al. (2021)
*Chlorella pyrenoidosa*/*Chlamydomonas spp.*	Microalgae	Pharmaceuticals (e.g., antibiotics), endocrine disruptors	Biotransformation/biodegradation and sorption	Shown to degrade or reduce concentrations of certain antibiotics and emerging pollutants under controlled conditions	Li et al. (2022), Gondi et al. (2022)
*Porphyridium cruentum*	Red microalga	Textile dyes, heavy metals	Extracellular polysaccharide binding and chelation	Employed in dye and metal removal studies; EPS aids pollutant binding	Gondi et al. (2022)
*Ulva spp.*/*Ulva lactuca*	Macroalga	N, P, some heavy metals	Ion exchange, bioaccumulation (coastal bio-filtration/IMTA)	Used in integrated multi-trophic aquaculture (IMTA) to remove nutrients and reduce eutrophication risk	Gengiah et al. (2023)
*Gracilaria spp.*/*Gracilaria verrucosa*	Macroalga	Heavy metals (Hg, As)	Cell wall polysaccharide biosorption	Demonstrated heavy metal uptake capacity, useful in coastal remediation and biomass valorization	Jeyadharmarajan and Gopalakrishnan (2023)
*Sargassum spp*. (beachcast)	Brown macroalga	Oil residues, hydrocarbons, nutrients	Bio-filtration and adsorption; collection prevents coastal re-mineralization	Beachcast Sargassum can be harvested and processed to prevent local pollution and convert biomass to products	Orozco-González et al. (2022)
*Cladophora glomerata*	Green macroalga	Hydrocarbons, organic pollutants	Biofilm-mediated degradation and sorption	Useful in remediation of oil-polluted water and construction of biofilters	Boonprab et al. (2017)

## Conclusion

Algae represent a highly promising and adaptable biological resource capable of addressing some of the most pressing global sustainability challenges. This review has demonstrated their wide-ranging applications in mitigating climate change through carbon sequestration, improving water quality via nutrient and pollutant removal, generating renewable energy in the form of biodiesel and bio-lipids, and enhancing food and feed security through nutrient-dense biomass. The integration of algae with waste-derived inputs—such as abattoir wastewater digestate and crude glycerol—further strengthens their role in promoting circular bioeconomy practices. For example, *Chlorella sorokiniana* has shown notable success in phycoremediation and high-quality biodiesel production under these conditions, and the use of macroalgae like *Asparagopsis taxiformis* in livestock feed has demonstrated significant reductions in methane emissions, offering a viable approach to climate-resilient agriculture.

Despite these promising developments, several key challenges continue to hinder the large-scale deployment and sustainability of algae-based technologies. High operational costs, especially those related to cultivation, harvesting, and drying, remain a major obstacle to economic feasibility. Additionally, the scalability of algae systems is constrained by contamination risks and the technical complexity of maintaining monocultures. Regulatory limitations, particularly regarding the approval of algal species for use in food and feed, further restrict commercialization and international market expansion. The lack of harmonized policy frameworks across regions adds another layer of difficulty to widespread adoption.

To fully harness the potential of algae in global sustainability efforts, future research must prioritize several critical areas. These include the development of cost-effective cultivation methods using low-cost or waste-derived media, as well as advances in genetic and metabolic engineering to enhance algal productivity and resilience. Comprehensive life cycle assessments and sustainability evaluations are essential to validate the environmental benefits of algal technologies at scale. Moreover, integrating algal systems into broader industrial symbiosis models—such as those involving agricultural and municipal waste—could increase efficiency and reduce costs. Equally important is the need for regulatory reform and harmonization to support commercialization, along with initiatives to improve public awareness and acceptance of algae-based products in food and agriculture. Addressing these issues will be essential for unlocking algae’s full potential as a cornerstone of sustainable development.
